# An analysis of Pompe newborn screening data: a new prevalence at birth, insight and discussion

**DOI:** 10.3389/fped.2023.1221140

**Published:** 2024-01-08

**Authors:** Ryan Colburn, David Lapidus

**Affiliations:** ^1^ odimm inc., Los Angeles, CA, United States; ^2^ LapidusData Inc., Oklahoma City, OK, United States

**Keywords:** Pompe (glycogen storage disease type 2/II), rare disease epidemiology, newborn screening (NBS), lysosomal storage disorder/disease (LSD), autosomal recessive inheritance, genetic prevalence, gnomAD, diagnosis/diagnostic yield

## Abstract

This study includes over 11.6M newborns screened (NBS) for Pompe Disease (PD) from 29 distinct universal screening programs across 8 countries and 4 continents. The birth prevalence of PD is 1:18,711, with no evidence of difference across populations of European, Latin American, or Asian ancestry, though differences may exist for PD subtypes. This study also compares these results, based on direct detection of disease and analyzed using a binomial method along with power analysis, with other methods for estimating the ‘frequency’ of rare genetic diseases (such as utilizing Hardy-Weinberg equilibrium on allele frequency and confidence interval analysis). This comparison demonstrates the implications of sample size and frames a discussion on its influence on the reliability of results when extrapolating to a population beyond the study dataset.

**Objectives:**

Primary: Establish a new figure for prevalence at birth for Pompe disease by collecting and analyzing the largest relevant dataset to date and using that result to project population prevalence at birth in a novel way. Secondary: Compare these results to previous analyses to offer a framework for evaluating ‘frequency’ data that can be applied to other rare, genetic diseases, along with methods to assess quality of estimates.

## Introduction and background

Pompe disease (PD), also known as glycogen storage disease type II, is a rare genetic disorder with an autosomal recessive inheritance pattern and a metabolic consequence. The acid alpha glucosidase (*GAA*) gene on chromosome 17 codes for production of the acid alpha glucosidase (GAA) enzyme/protein which is responsible for a step in the process of breaking down stored energy in the lysosome on its way to a usable form (energy stored as glycogen breaks down into glucose). Single or multi-nucleotide sequence variants within this gene can have a range of impact on the resultant enzyme produced: from minimal functional change to decreased functionality of the enzyme to reduced quantity of functional enzyme produced (as low as zero). When function- or quantity-limiting variants are present on both inherited *GAA* alleles, leading to sufficiently reduced GAA enzyme activity, Pompe disease is diagnosed. With decreased functional enzyme, glycogen or partially broken-down glycogen accumulates in the lysosome, initiating a metabolic cascade which eventually leads to cellular damage. While nearly every cell contains lysosomes which have the potential to be impacted by this disorder, cell damage is primarily observed in specific cells which rely on this stored energy more heavily (for example proximal skeletal muscle). Within the 18,000+ nucleotide long *GAA* gene, hundreds of variants have been established as pathogenic (1) for PD thus far. There is some ability to predict the impact on GAA enzyme based on the variant type and location. Historically, the disease has been categorized into subgroups based on an “age of onset” classification: initially as infantile, juvenile, and adult subgroups and more recently, an infantile onset form (IOPD) and a late onset form (LOPD). These age-of-onset based classification systems can be misleading as even persons diagnosed as late onset may have (had) detectable disease features in infancy. A 2010 analysis detected enlarged lysosomes in a one-month-old “asymptomatic” newborn who had been diagnosed via newborn screening (NBS) with “milder late-onset” genetic variants (2). A 2017 study of seven infants diagnosed with LOPD via NBS concluded that all seven patients demonstrated Pompe specific signs of motor involvement by age six months (3). Of note, four of the seven were identified as having a homozygous c.-32-13T>G genotype, which is predicted to have the “mildest” associated PD phenotype. A more-recent 2022 study of 20 infants diagnosed with LOPD via NBS also found that the entire cohort had detectable features of Pompe within the first years of life—including the four (20%) homozygous c.-32-13T>G participants (4). These observations reinforce the importance of considering an alternate basis for a classification system. A diagnosis of Pompe disease, having a spectrum of progression, may be sufficient, with a severe subcategory as a pragmatic distinction for cases with uniquely urgent sensitivity to starting treatment. This severe PD subset would encompass the generalized description of IOPD as those with zero or near-zero GAA enzyme function, who may have cardiac involvement, as there is an expectation of rapid accumulation of potentially irreversible progression in such cases where treatment is unavailable or delayed. That notwithstanding, this study uses the IOPD and LOPD distinctions as provided by the contributing NBS programs, while the discussion considers the generalizations as described.

Understanding disease frequency^1^ (incidence^2^ and prevalence^3^) has important implications. These values inform public health impact, assist in identification of affected communities, and support the development of disease-specific therapies. Because of the importance of epidemiology to rare diseases, estimates must be generated, revisited, and challenged. In the case of Pompe disease, increasing adoption of NBS affords us an opportunity to improve estimates of PD birth prevalence.

**Figure 1:**
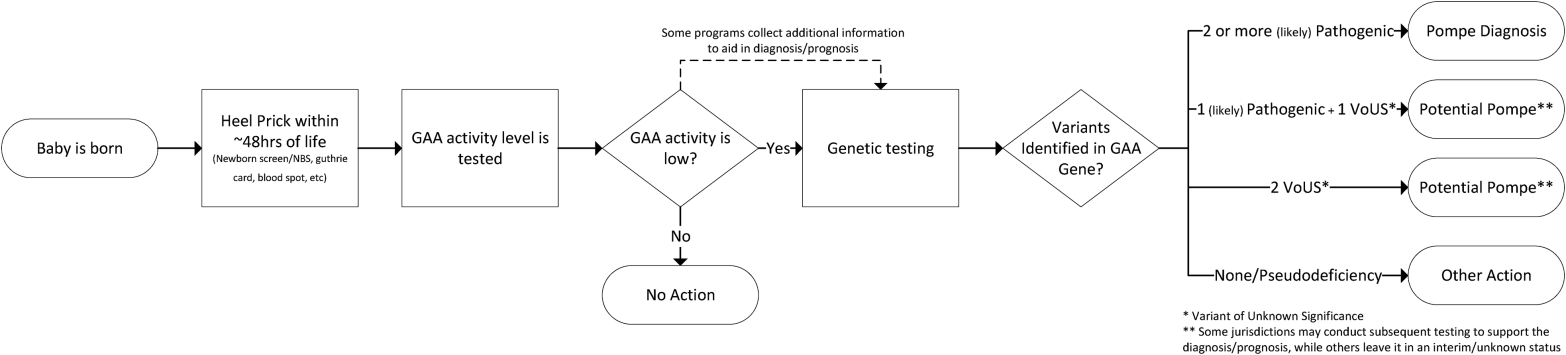
Generalized Pompe diagnosis via NBS process flow.

This paper uses the diagnosis criteria of the NBS programs that supplied the data which represents a diagnostic threshold of both a biochemical screen and a molecular (genetic) confirmation. Summarily, individuals included in the analysis have been assigned a diagnosis of PD based on low GAA enzyme activity *and* two (or more) identified pathogenic or likely pathogenic variants to the *GAA* gene. Because the diagnostic algorithms used in NBS provide visibility into the biochemical consequence of the detected variants already, testing to determine configuration of the variants (trans vs. cis) is not routine for diagnosis. There are circumstances where additional testing may be prudent. While each screening jurisdiction has its own detailed NBS workflow, a generalized process flow for diagnosis of Pompe via NBS is shown in Figure 1.

**Figure 2:**
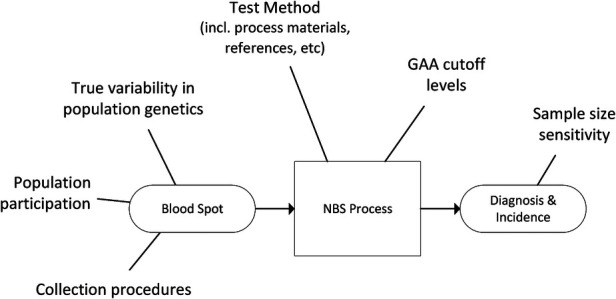
Generalized example of sources of variability in NBS based prevalence estimates.

A general example of sources of variability for the NBS data used in this study is provided in Figure 2.

## Methods

### Data collection

A literature review of available NBS data accounted for approximately 4 million newborns universally screened for PD (i.e., not selectively screened or high risk). To estimate the completeness of these published figures, a comparison was made against the estimated number of births in jurisdictions that have described their NBS programs, factoring in the time frame for active screening and birthrates for that jurisdiction. This analysis suggested that results for fewer than half of total Pompe screened births had been published. To resolve this gap, and improve the size and representativeness of the analysis, an attempt was made to collect the missing US data from the publicly funded NewSTEPs data repository, but the data were not made available for this study. As a result, individual states and countries (departments of health, newborn screening programs, etc.) were contacted by one of this paper's authors (RC), to gather additional data as well as verify previously published data. Each program provided the following variables which were included in the analysis: date range, birth count, diagnosed count and any sub or adjacent category of diagnosed count (e.g.: IOPD, LOPD, unknown disposition). These efforts increased the dataset to over 11.6M newborns.

### Analysis

The initial analysis uses the traditional approach of using a 95% confidence interval (CI) to estimate birth prevalence from individual and aggregate datasets - in this case, specifically the Wilson methodology which is appropriate for very small proportions (frequencies of occurrence). A limitation of this approach is that it considers only the sample size and proportion of occurrence for the discrete set of data used. For epidemiological analyses, it can be useful to consider clusters of discrete data or subsets of general data, which are intended to represent a hypothesized feature of interest, for example geography (by region, continent, etc.). With a traditional 95% CI approach, it is necessary to do this by creating subsets and groupings of the data (for example, different geographical regions)^4^. In this approach, these aggregate datasets contain all the elements of the source datasets, but any unique characteristics (such as birth prevalence for an individual contributing jurisdiction) become indistinguishable when aggregated. This approach becomes challenging as the amount and diversity of data, or number of clusters of interest, increases. It becomes difficult to interpret the discrete analytical outputs, and more data preparation is required. These factors may discourage the kind of exploration that drives the development of new knowledge.

This paper uses a methodology that can address these limitations. We recognize a benefit to retaining the unique features of a source dataset (in this case birth prevalence within an NBS jurisdiction), but also acknowledge heredity as a process common to all humans, albeit with potential factors that may differentially impact sub-populations. We maintain both features by utilizing a binomial methodology, a technique often used in the field of process analysis, which has mathematical terms that factor both individual/contributing datasets and the total dataset in a single analytical output. Additional detail on the supporting calculations can be found in Appendix A.

A visual comparison of the two approaches (Wilson 95% CI and binomial) is shown in the Results section. The birth prevalence value is the same for both approaches^5^, but the organization of the binomial model yields additional observations which will be covered in the Discussion section.

## Results

Pompe disease birth prevalence is 1:18,711 births (5.3 per 100,000 births) in this dataset of over 11.6M newborns screened for Pompe across 22 states and 8 countries on 4 continents between 2010 and 2022.

Of the 11,619,662 screened newborns included in this analysis and the 621 total Pompe diagnoses, 91 were classified by their NBS programs as IOPD (1:126,118 or 7.9 per 1M births), and 524 as LOPD (1:21,902 or 45.6 per 1M births), while 6 were only classified generally as PD without subtype distinction (5 from Taiwan, 1 from Mexico). These figures exclude an additional 120 newborns who were flagged as having low GAA enzyme activity and identified *GAA* variants, but with interim “unknown” dispositions. These are potentially LOPD or pseudodeficiency cases, but with previously uncharacterized genetic variants, or cases awaiting further reporting at the time of data collection. When states/countries identified an “unknown” diagnosis, these specific counts were not included in the analysis, but they're noted in the data table which can be found in Appendix B. If any of the 120 “unknown” cases are ultimately diagnosed as PD, then the true birth prevalence of PD is higher than the 1:18,711 reported here^6^. These additional cases are not likely to be severe PD, because the very low-to-no enzyme activity and typical cardiac involvement associated are identifiable features that can support an IOPD diagnosis. Among the sources of variability summarized in Figure 2, there are two noteworthy examples that further suggest this analysis as an underestimate of birth prevalence: Some cases have been lost to or refused follow up, and some NBS jurisdictions use enzyme activity cut-offs that are too low to identify all cases of PD^7^ (5). The approach of this paper, to only use cases diagnosed by NBS, is conservative as the examples of excluded uncertainty above can only increase the prevalence of Pompe as they would add, not subtract cases. The resultant birth prevalence value is therefore confidently a minimum estimate, i.e., the true birth prevalence is at least 1:18,711.

**Figure 3:**
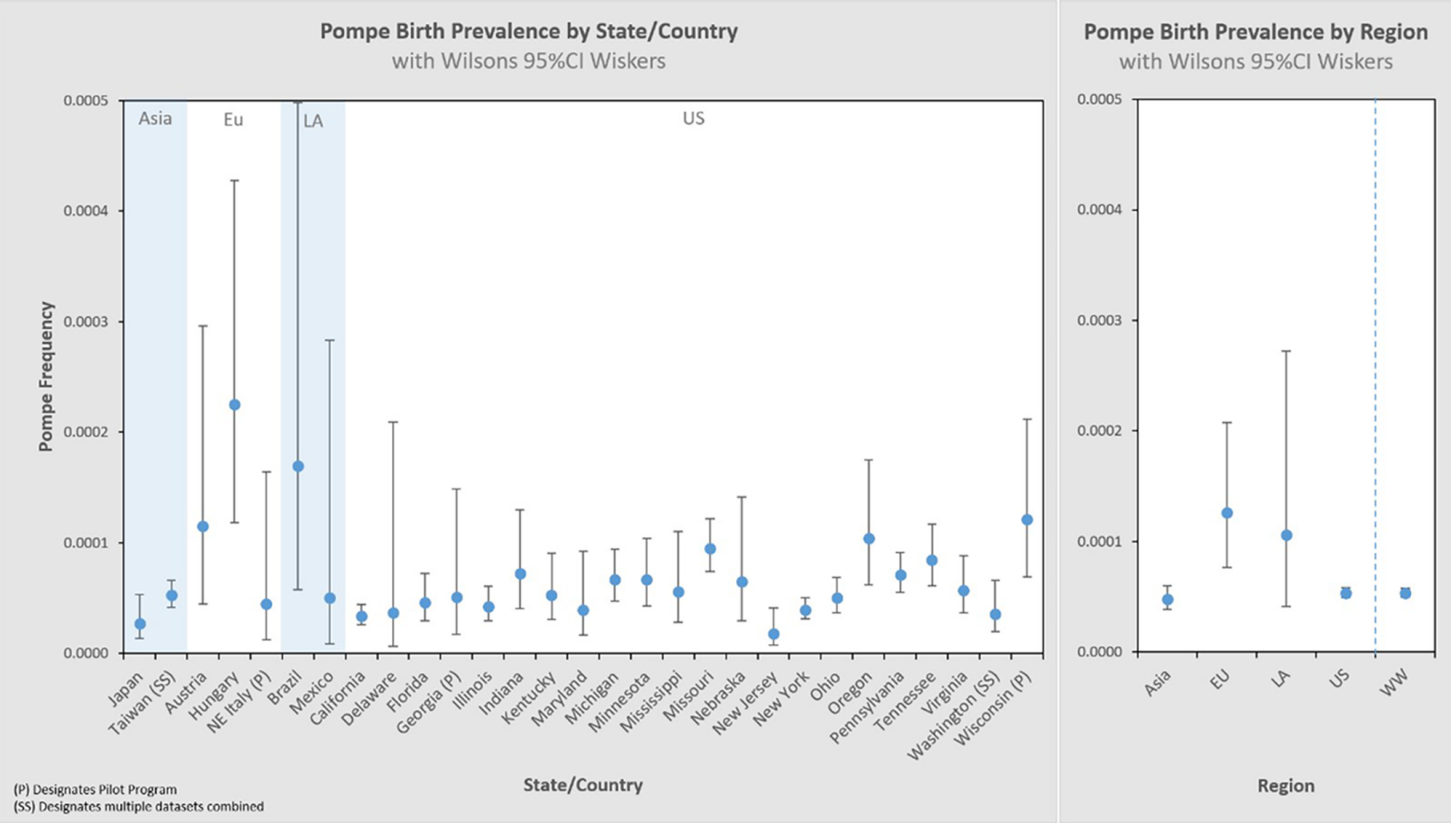
Pompe birth prevalence by state/country and by Region with 95% CI.

Figure 3 presents these results in a typical format for “frequency” analyses. Each jurisdiction's observed birth prevalence is shown along with a 95% CI, as well as several regional groupings [Asia, Europe (Eu), Latin America (LA), United States (US), and worldwide (WW) aggregate]. In each of these figures, birth prevalence is shown as a point, and the confidence interval shown as whiskers. When comparing each value with another, the more the confidence interval (whiskers) overlaps, the lower the statistical likelihood that a difference exists between them.

**Figure 4:**
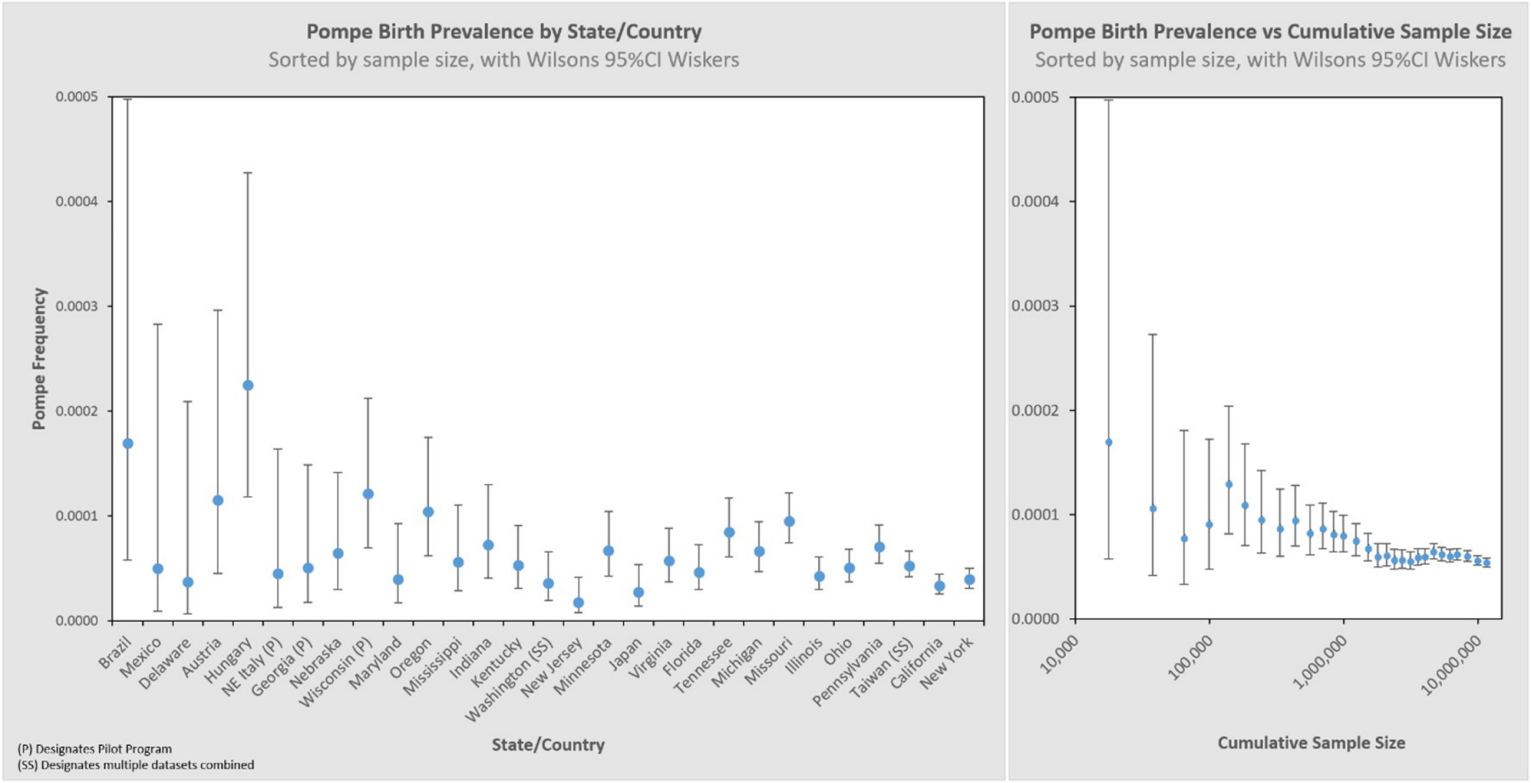
Pompe birth prevalence by state/country with 95% CI, and cumulative rate ordered by newborns screened (increasing sample size).

Larger data sets have greater statistical precision (narrower whiskers), which is one characteristic by which to judge the reliability of the result. We can visualize this by organizing the same dataset by sample size, shown in the left panel of Figure 4. The sample size increases from left to right on the x-axis, and the corresponding 95% CI decreases (narrower whiskers). Another way to visualize this is on the right panel which cumulatively aggregates the birth prevalence results by jurisdictions of increasing newborns screened. When only the smallest data sets are considered (∼10k newborns) the calculated birth prevalence is so imprecise as to be barely usable for public health purposes. The addition of increasingly larger samples (moving along the x-axis to over 10M newborns) quickly leads to leaps in precision. This can be seen in this figure where the upper bound value of the 95% CI range on the left is 8.6× the lower bound value of the 95% CI range, while on the right it is only 1.2×. It is also notable that there are diminishing returns along the x-axis—after a certain point, even hundreds of thousands of additional newborns have little impact on precision (the spread of the whiskers).

**Figure 5:**
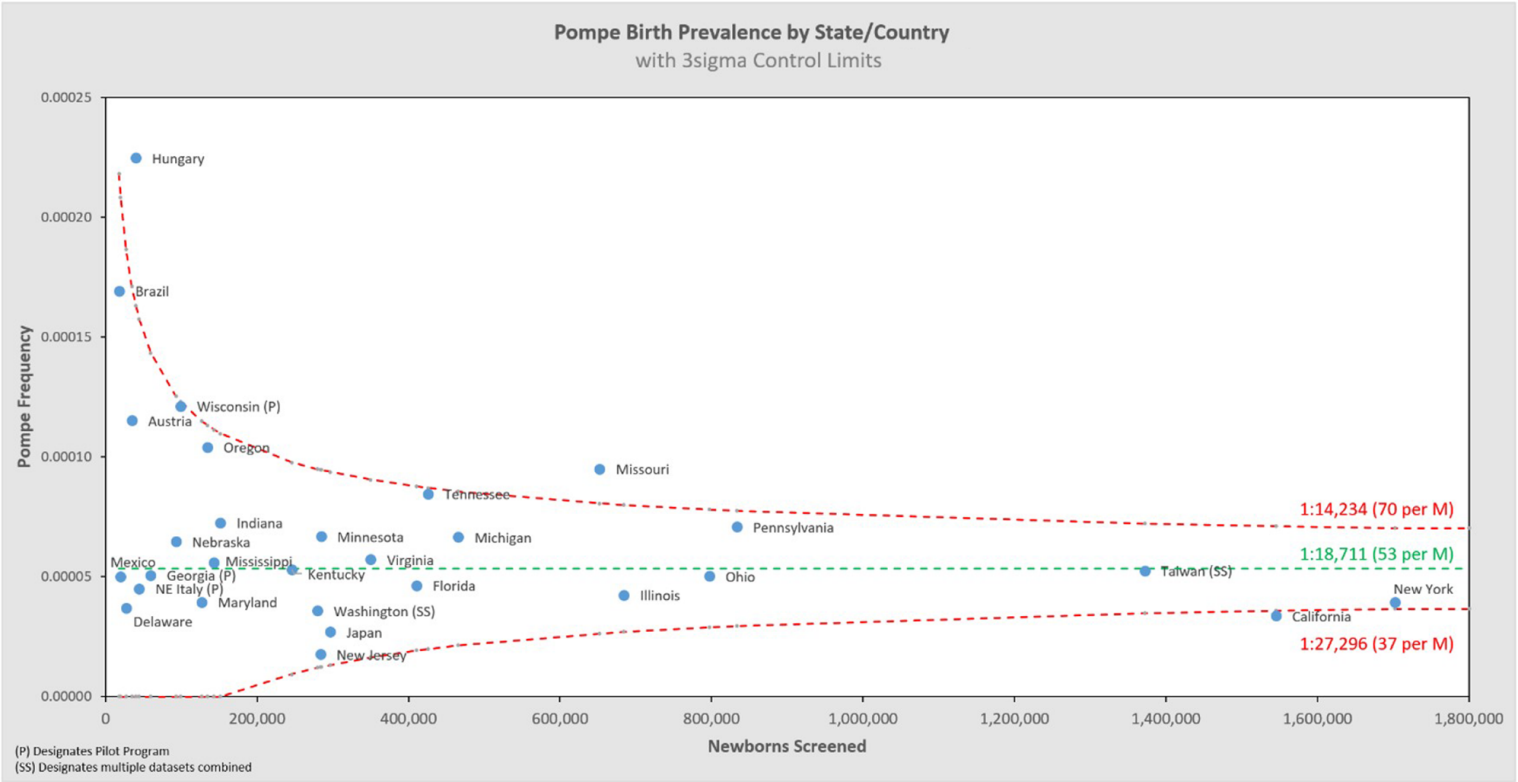
Pompe birth prevalence vs. newborns screened by state/country.

**Figure 6:**
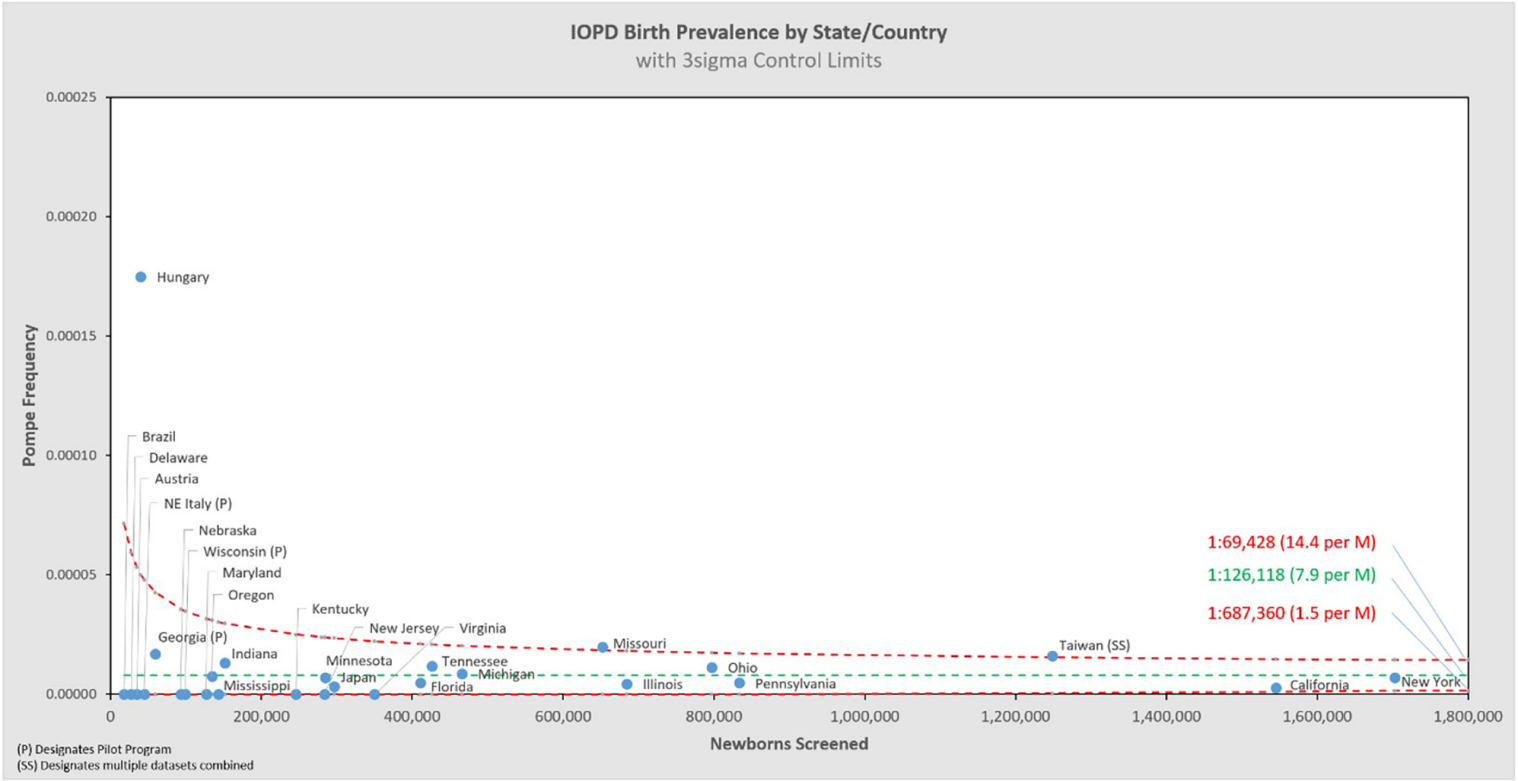
IOPD birth prevalence vs. newborns screened by state/country.

**Figure 7:**
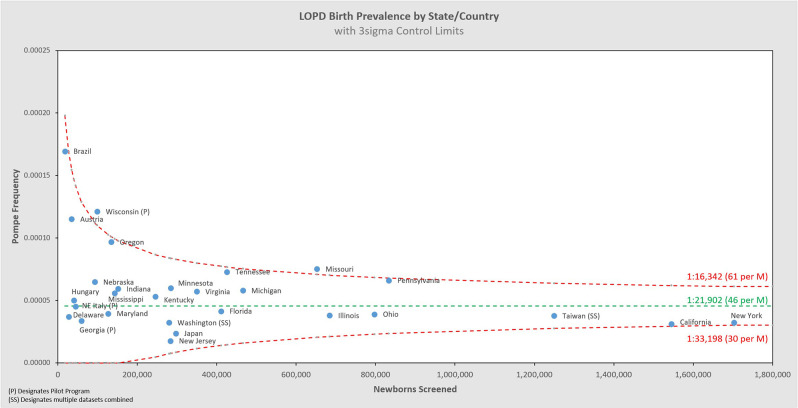
LOPD birth prevalence vs. newborns screened by state/country.

Figure 5 shows the same data analyzed using a binomial model, and Figures 6, 7 break out IOPD and LOPD respectively. In each of these figures, birth prevalence is shown as a point and the uncertainty figures (±3σ control limits that consider both the individual point as well as the entire dataset) are shown as connected lines through this value for each point. With this analysis one can visualize the number of newborns screened, the birth prevalence, and individual uncertainty relative to the entire dataset for each individual jurisdiction. Adherence of each jurisdiction to the narrowing uncertainty range suggests a convergence of birth prevalence as sample size increases.

**Figure 8:**
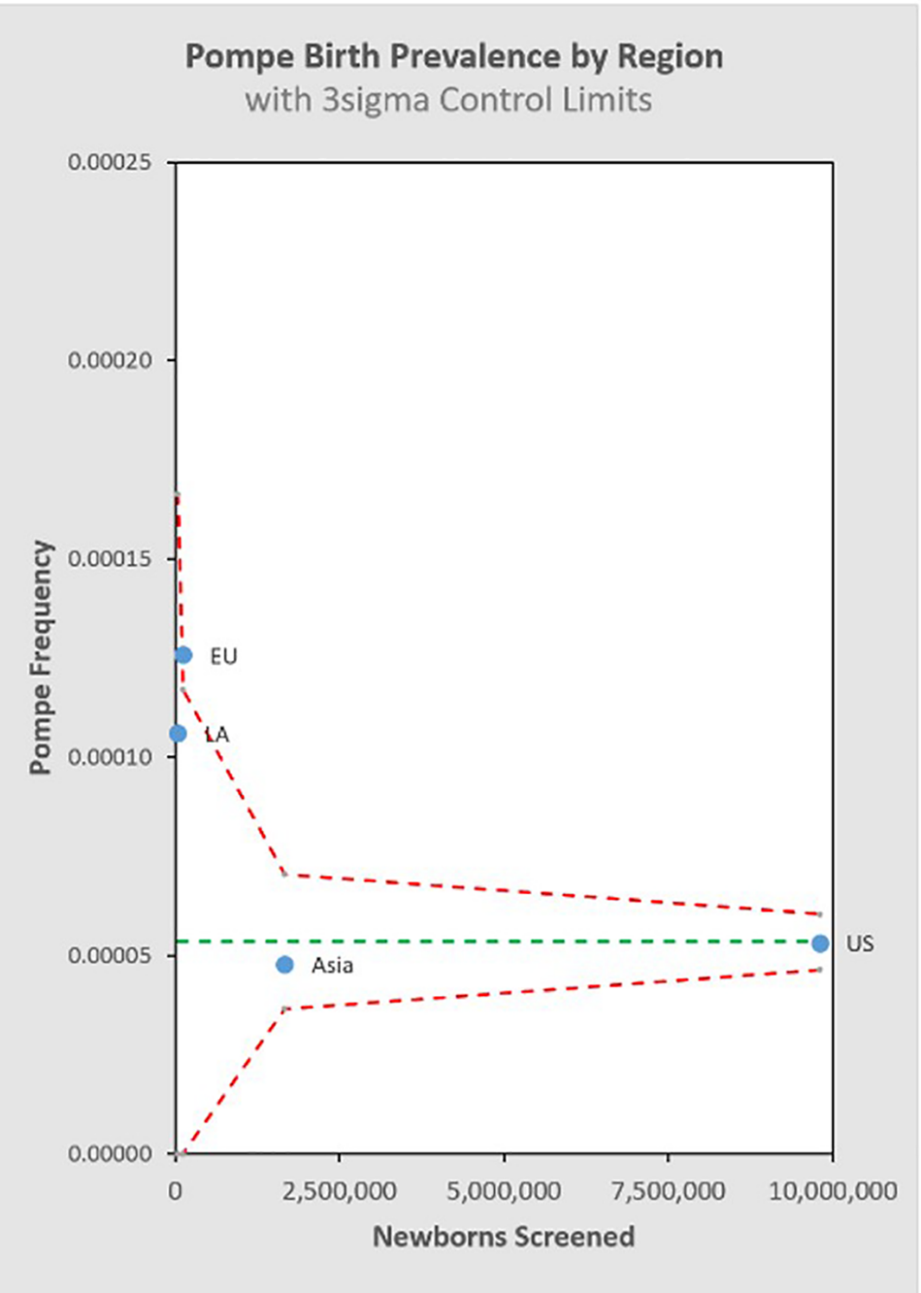
Pompe birth prevalence vs. total newborns screened by region

Figure 8 groups the State/Country data by common regional groupings.

## Discussion

The birth prevalence of PD for the 11,619,662 newborns screened included in this dataset is shown in Table 1, along with population birth prevalence projection ranges produced through two different analytic approaches. The birth prevalence value is the same in each method, but each has a different uncertainty range due to slightly different techniques for incorporating statistical uncertainty. An additional note on the standard values used: ±3σ is a higher statistical threshold (inclusive of more possible outcomes) accounting for 99.7% of observed normal variation, which is more rigorous than the 95% confidence interval, hence the broader range than the 95% CI.

**Table 1:** Measured Pompe birth prevalence with two projections for population birth prevalence range.

	Measured birth prevalence	Projected population birth prevalence	
	Direct detection	Wilsons 95% CI	±3σ control limit convergence
Total Pompe	1:18,711^a^ *53.4 per 1M births*	1:20,242 , 1:17,296 *49.4–57.8 per 1M births*	1:27,296 , 1:14,234 *36.6–70.2 per 1M births*
IOPD	1:126,118 *7.9 per 1M births*	1:154,829 , 1:102,731 *6.5–9.7 per 1M births*	1:687,360 , 1:69,428 *1.5–14.4 per 1M births*
LOPD	1:21,902 *45.6 per 1M births*	1:23,859 , 1:20,105 *41.9–49.7 per 1M births*	1:33,198 , 1:16,342 *30.1–61.2 per 1M births*

^a^ Using the Hardy–Weinberg principle, this projects that 1:68.9 people are carriers of a Pompe contributing genetic variant.

This study is the most comprehensive examination of birth prevalence to date, and its results address the question of whether there are significant differences in prevalence across different ancestry groups: there is little evidence to support that significant differences in total PD birth prevalence exist across the jurisdictions included. It remains possible that differences may emerge with even larger and broader data sets, but in the absence of further evidence, it is appropriate to apply the results from this study for total PD birth prevalence to any given population.

The direct detection of PD biomarkers used in the NBS diagnosis process offers a significant step forward for estimating PD birth prevalence compared to data collected via indirect methods, such as carrier screening which estimates prevalence at conception. Additionally, this dataset is 3–4 orders of magnitude larger than the indirect datasets previously used, and it is generated from more a broadly representative population. There is still inherent bias to this dataset, which will be discussed below, but on balance this dataset represents a major advance in Pompe disease epidemiology. These factors favor the total PD birth prevalence from this study as the basis for population-wide prevalence projections as a replacement for previous estimates.

### Reflections on previous work and responsibility in interpretation

Groft & de la Paz (6) give an overview of the impact that epidemiological data can have on evolving knowledge, enabling movement from misperceptions and myths toward scientific realities. They describe the importance and implications of accurate epidemiological data for the study of rare diseases. The data used in this study represent progress toward a true PD prevalence, along with a statistical model which can gauge whether a given data set is sufficiently large to represent true population prevalence. We further propose the use of statistical power^8^ as a tool to estimate the strength a given study relative to the misperception/myth vs. scientific realities/truth continuum.

The development of new knowledge around a topic is fundamentally iterative. It is essential to regularly revisit and challenge the assumptions, foundations and context from which early knowledge and conclusions are derived.

Two commonly referenced studies of Pompe frequency, from 1998 and 1999, use the carrier-frequency method, based on allelic prevalence, to estimate Pompe prevalence at conception (7, 8). Both estimate prevalence at 1:40,000 as an update from an even older 1:100,000 estimate. Martiniuk et al. analyzed 928 randomly selected individuals from New York to establish a carrier frequency of 7 known pathogenic variants in the *GAA* gene, with these variants seen in 29% of a diagnosed reference dataset. In a separate effort, Ausems et al. analyzed 3,043 randomly selected blood spot cards from various regions of the Netherlands to establish a carrier frequency of three known pathogenic variants in the *GAA* gene. These three variants were seen in 63% of the then diagnosed Dutch PD population. Ausems et al. stated that their higher variant representation (63% vs. 29%) led to ‘extrapolations that were considerably more accurate’ than the approach of Martiniuk et al., and that it was merely coincidence that both studies estimate the same point value for disease frequency.

Both papers noted the limitations of their estimates, including the statistical uncertainty inherent in their small sample sizes. Despite this acknowledged uncertainty, let alone the still-unknown uncertainty, the cross-validation effect of these two studies has led to a broad, highly entrenched adoption of 1:40,000 as a projection of Pompe prevalence that continues to be widely circulated.

In 2021, Dr. Park published an analysis (9) that applied the same carrier-frequency method to a much larger and more broadly representative dataset. She also considered a larger set of PD variants. Park's dataset includes over 140,000 genetic screens, with sample diversity across eight identified demographic groups, and considers 154 pathogenic or likely pathogenic variants. Using this dataset, Park projects a prevalence (at conception) of 1:23,232; which is notably close to this paper's measured value for birth prevalence of 1:18,711.

The implications of Park's result being in proximity to the result of this study may be impactful for gaining further understanding of PD, as well as for the broader rare disease community.

With hindsight, it is clear that the two early studies were not so different as they must have seemed at the time, and the underlying strength of their datasets was weaker than the 20+ years of use would suggest. A power analysis, using only information available at the time of each study (namely sample size and an estimation of proportion/frequency of occurrence), provides a confidence figure for the results of these two studies. Table 2 shows a summary of inputs and the results from a power analysis, along with the more recent Park study, and this paper. The associated power curves for each study, along with a visual comparison of the uncertainty ranges from Table 2. are shown in Appendix C. In addition, rows three and four revisit assumptions made in the Ausems paper for additional perspective:

**Table 2:** Comparison of multiple datasets to project prevalence of PD.

Year / Method	Sample size	Variants covered	Effect measured?	Direct/indirect	Projection	*α* = 5% (95% CI)	*β* = 80% power range (with *α* = 5%)
1998 / Martiniuk	928	7	*N*	Indirect	1:32,189^a^ *31.1 per 1M births*	1:278,014 , 1:3,758 *3.6–266.6 per 1M births*	– , 1:1,137 *(0)−879.2 per 1M births*
1999 / Ausems	3,043	3	*N*	Indirect	1:40,059^a^ *24.9 per 1M births*	1:115,325 , 1:13,886 *8.7–72.0 per 1M births*	– , 1:2,209 *(0)−452.6 per 1M births*
1999 / Ausems (2022 updated asumptions)	3,043	3	*N*	Indirect	1:28,138^a^ *35.5 per 1M births*	1:76,270 , 1:10,294 *13.1–97.1 per 1M births*	– , 1:1,859 *(0)−538.0 per 1M births*
1999 / Ausems (Martiniuk approach)	3,043	3	*N*	Indirect	1:15,298^a^ *65.4 per 1M births*	1:30,743 , 1:7,631 *32.5–131.0 per 1M births*	– , 1:1,395 *(0)−716.6 per 1M births*
2021 / Park	141,456	154	*N*	Indirect	1:23,177^a^ *43.1 per 1M births*	1:25,368 , 1:21,176 *39.4–47.2 per 1M births*	1:232,558 , 1:10,030 *4.3–99.7 per 1M births*
2022 / NBS_Direct	11,619,662	Any/all^b^	Y (enzyme activity)	Direct	1:18,711 *53.4 per 1M births*	1:20,242 , 1:17,296 *49.4–57.8 per 1M births*	1:21,053 , 1:16,779 *47.5–59.6 per 1M births*

^a^ Note these figures are different than reported in their respective papers. They are recalculations based on the inputs provided in those papers.

^b^ Bounded by gene sequencing method and procedures of the responsible lab.

• Row 3: The original Ausems analysis excluded homozygosity of a mild variant (c.-32-13T>G) it considered on the grounds that it probably did not give rise to Pompe. The cited studies by Rairikar et al. and Huggins et al. compel a reversal of this exclusion, which is reflected in row three.
• Row 4: Ausems’ original analysis used separate calculations for IOPD and LOPD carriers, whereas Martiniuk considered carriers of any pathogenic variant as a single cohort without a homozygous mild variant exception. Applying Martiniuk's approach that includes scaling for the variant coverage estimate of the time, stated at 63% for Ausems, yields the projection provided in row four.

The utility of a power calculation is to show how narrowly a range can be defined while remaining confident that we do not exclude the truth from that range. As applied here, the calculation is based on the sample size of a given study, the observed prevalence in that study, and a power value, β, that can be selected for the purpose (common values for β are 0.8 or 0.9). The calculation yields a “power curve,” which illustrates a range of possible results for which the study is adequately powered to reject that the true prevalence is outside of this range with a likelihood of β (for example, 0.8 or 80%). In simpler terms, if a study were performed 100 times with the same parameters, these power ranges are the narrowest 95% CI range that 80 of those 100 repeats would fall into.

### Accelerating learning | reflections on effort, building momentum and a validated reference concept

The analytic approach used in this study could be applied to other conditions, but the process for collecting the data is not scalable in a reasonable timeframe. While it is currently time consuming to gather NBS data, the real challenge is the massive undertaking required to implement NBS for a condition. As evidence of this point, PD was first submitted for consideration on the Recommended Uniform Screening Panel (RUSP) in the US in 2006, but it wasn't included on the RUSP until nine years later when it was approved in 2015. As of September 2023, 45 states (including DC) accounting for approximately 87% of US births had initiated a Pompe NBS program, while just over 2% of worldwide births have programmatic/universal NBS for Pompe. It is not tenable to expect a 15+ year process for each of the projected 10,000+ rare diseases (10) to get to the point of having reliable epidemiological data—our approach to these challenges must evolve.

The availability of increasingly large genetic datasets that are publicly accessible (for free), such as that used by Park^9^, point the way forward for some diseases. The convergence of Park's estimate (using expanding broad general open access datasets), towards this study's result (using data from a major effort specific to a single disease) is exciting because it supports the use of genetic databases where NBS data are not available. It suggests that publicly available genetic data may have sufficient sample size and diversity to be useful for other conditions. Replicating these two studies for other conditions where NBS is already in place can further delineate the strengths and weaknesses for use of publicly available data. This validation approach can cut years, if not decades, from the process of generating quality epidemiological insight for other diseases. It is worth emphasizing that this type of progress acceleration depends on the availability of datasets.

It is important to highlight some key considerations and limitations of this approach that could affect other diseases^10^:

• The disease considered here is monogenic with an autosomal recessive inheritance pattern.
• Some knowledge of pathogenic variants is established (or can be reasonably projected).
• Appropriate sample size is determined by rarity, so conditions that are rarer than PD will have lower statistical power. This improves as genetic databases increase in size.
• The genetics of the sample population may be a source of bias. For this analysis of PD, both the NBS dataset and the publicly available genetic datasets may not necessarily represent the population of interest for all research questions. This factor improves as datasets increase in diversity and representation.

### Possible regional variation in PD subtypes

In general, we can look at the R-Value of a linear regression of sample size vs. birth prevalence for the regions included in the analysis to suggest that this single variable model explains as much as 81.6% of the observed variability between the individual screening jurisdictions (Appendix D). This metric shows that differences between regions are small relative to the effect of sample size itself for the NBS programs considered in this analysis. It is interesting to note that while the total birth prevalence seems to converge towards single figure globally, there does appear to be a meaningful difference in the IOPD/LOPD breakdown in specific regions. In the US, 12% of NBS cases are diagnosed as IOPD and 88% LOPD (this likely underestimates the share of LOPD, as “unknown” cases are expected to skew towards LOPD). By contrast, in Asia (primarily Taiwanese data) the diagnoses are 28% IOPD and 72% LOPD. One possible explanation is the “mild” c.-13-32T>G variant that is common in the US population, but not in the Taiwanese population. It is considered a “rescue” variant because it results in enzyme with enough function to shift towards a LOPD phenotype even when paired with a severe variant that would otherwise suggest IOPD. This observation that total PD birth prevalence is generally similar across populations, while the distribution of IOPD vs. LOPD differs, may be of interest in an investigation of survivability and modifiers for propagation of recessive conditions on an evolutionary scale. Additionally, while the data considered in this analysis suggests convergence of total PD birth prevalence, even across diverse populations, the authors note that data from the two most populous countries (China and India) are not directly represented, which may be a source of variability when projecting global prevalence.

### An example of applied insight towards the topic of diagnosis timelines and diagnostic delay

It is clearly desirable to diagnose people who have a potentially severe yet treatable condition such as PD in a timely manner. For many rare diseases, the term “odyssey” is applied because it can take years or even decades to get a diagnosis, as noted by Groft and de la Paz. Surveys are often used as a retrospective estimate of time-to-diagnose as a performance metric for the clinical diagnosis process. This study offers insight on this same concept in a less subjective way: In April 2022, Sanofi presented to the AMDA (Acid Maltase Deficiency Association) that the Pompe Registry had 819 PD diagnosed participants in North America (an alternate, more conservative figure for this is 343 provided by Reuser et al. (11)) — not a perfect estimate of diagnosed prevalence, but, reasonable as an order of magnitude estimate. We can compare this with an estimated North American prevalence of over 23,000 (using LOPD birth prevalence only, as a simplification based on high mortality in undiagnosed/untreated severe PD) to show that <5% of the Pompe population are represented in the registry. While some diagnosed people do not participate in the registry, the rest of the gap must be due to under/misdiagnosis. This suggests that a small fraction of the global Pompe population has been diagnosed. This helps quantify the inefficiency of the clinical diagnosis process for this treatable condition, with the suggestion that those with public health or commercial interests should consider strategies that increase emphasis on higher-yield diagnostic processes, such as NBS. The implications of a strategy to improve diagnostic yield would be impactful on further development of knowledge, through study participation, participant/spectrum diversity, business case justification, etc.

### Post Factum

While working through publication of this manuscript, additional data were published about incidence of PD in Italy (12), as well as updated data made available by the Tennessee Department of Health NBS program, and data shared by both Colorado newborn screening program and North Carolina department of health and human services via posters at the APHL/ISNS NBS Symposium in Sacramento, October 15–19, 2023. An updated binomial analysis, inclusive of this additional data is available in Appendix E. In short, these additional data fit very well within the model presented. Summary stats with this additional data included: 12,060,529 newborns screened for PD with a birth prevalence of 1:18,698.

## Data Availability

The original contributions presented in the study are included in the article/Supplementary Material, further inquiries can be directed to the corresponding author.

## Footnotes

^1^Frequency is a catch-all term that captures any measure of disease occurrence in a population.

^2^Incidence measures the number of new cases that occur in a specified time period (e.g., cases per year). Colloquially, it is often used to mean “prevalence at birth”. However, prevalence at birth is not actually a measure of incidence because this metric is independent of time (e.g., cases per 100,000 births).

^3^Prevalence measures the number of affected persons among a broader population. For example, birth prevalence measures the number of affected newborns out of the total newborn population. A related metric is prevalence at conception (familiar as the result of the Hardy–Weinberg formula). It differs from birth prevalence as some pregnancies do not reach term. Compared to birth prevalence, total population prevalence may be lower due to disease-related mortality. As mortality decreases (e.g., from timely access to quality treatment), prevalence converges towards birth prevalence.

^4^To consider relationships between regional datasets, one would compile several aggregate datasets (i.e., sample 1, and separately a dataset of sample 1 + sample 2, and separately still a dataset of sample 1 + sample 2 + ... + sample *n*.

^5^The projected uncertainty ranges are different (but concentric). They are calculated differently, with different specific meanings in each approach, but can generically be interpreted as a “variability figure”.

^6^If all 120 Unknown cases resolved in a diagnosis of Pompe, then the birth prevalence for this dataset would be 1:15,681 (63.8 per 1M births).

^7^For example: Between 8/1/17 and 7/31/21, Minnesota Department of Health had one case lost to follow up which it considers to be LOPD, but is not included, and two confirmed LOPD cases which were not identified by NBS due to low screening cutoffs. They were instead identified as a result of familial testing after a later-born sibling was identified. These cases were not included in this study. Differences in enzyme activity thresholds may also explain some variability between jurisdictions.

^8^In Statistics, Power (β) is the probability of avoiding a Type II error—i.e., correctly rejecting the null hypothesis. It can be used to assess how confident one can be based on the input parameters available by providing the probability of correctly detecting a difference from a projected prevalence based on how big that difference is.

^9^The exception in her study is Pompe Registry data (used as a part the variant cross-checking process she described), some of which is available through publication, but is ultimately owned and siloed by the pharmaceutical sponsor.

^10^This is an incomplete list of limitations to consider.

## Appendix A

### Equations used in the Wilsons CI analysis

Each Plotted point represents the proportion of occurrence for the specified cluster

$$p_{c}=\frac{x_{i}+\ldots +x_{n}}{n_{i}+\ldots +n_{n}}$$

 Where,

 *p_c_* is the proportion of occurrence for the cluster

 *x_i_* is the count of occurrence for the subgroup

 *n_i_* is the size of the subgroup

Each Plotted whisker represents the CI range for the specified cluster

$$CI=\;\frac{\;p_{c}+\frac{z^{2}}{2n_{c}}}{1+\frac{z^{2}}{n_{c}}}\pm \frac{z}{1+\frac{z^{2}}{n_{c}}}\sqrt{\frac{\;p_{c}(1-p_{c})}{n_{c}}+\frac{z^{2}}{4n_{c}^{2}}}$$

 Where,

 *p_c_* is the proportion of occurrence for the cluster

 *z* is the *z*-score associated with the desired CI For a 95% CI, a *z* score of 1.96 is used.

 *n_c_* is the size of the cluster

### Equations used in the binomial analysis^11^

Each plotted point represents the proportion of occurrence for the specified subgroup

$$p_{i}=\frac{x_{i}}{n_{i}}$$

 Where,

 *p_i_* is the proportion of occurrence for the subgroup

 *x_i_* is the count of occurrence for the subgroup

 *n_i_* is the size of the subgroup

The centerline represents the average proportion of occurrence for the entire dataset

$$\bar{\;p}=\frac{\sum x_{i}}{\sum n_{i}}$$

 Where,

 $\bar{\;p}$ is the calculated average proportion for the entire dataset used

 *x_i_* is the count of occurrence for the subgroup

 *n_i_* is the size of the subgroup

The Control Limits (CL), or expected range, are shown as connected lines through each individual subgroups ±3σ proportion of occurrence

$$CL_{i}=\bar{\;p}\pm k\sqrt{\frac{\bar{\;p}(1-\bar{\;p})}{n_{i}}}$$

 Where,

 $\bar{\;p}$ is the calculated average proportion for the entire dataset used

 *k* is sigma value (3 is commonly used)

 *n_i_* is the size of the subgroup

 Note: lower control limit proportions are bound at 0, while upper control limits are bound at 1

## Appendix B

Table of input figures for each contributing region, along with source citation and date range references.

**Table:**

Region	State/Country	*N*	Unknown	IOPD	LOPD	Total Pompe	Total Incl. unknown	IOPD Prevalence_birth_ (1:X)	LOPD Prevalence_birth_ (1:X)	Total PD Prevalence_birth_ (1:X)	% IOPD	% LOPD	Data source	Published date	Data start date	Data end date
US	Illinois	684,290	8	3	26	29	37	228,097	26,319	23,596	10%	90%	Int. J. Neonatal Screen. 2020, 6, 4; doi:10.3390/ijns6010004	21-Jan-20	3-Nov-14	30-Sep-19
US	California	1,545,100		4	48	52	52	386,275	32,190	29,713	8%	92%	Genetic Disease Screening Program, California Department of Public Health	11-Jun-22	29-Aug-18	1-Mar-22
US	Wisconsin (P)	99,018		0	12	12	12		8,252	8,252	0%	100%	https://www.wiaap.org/pompe-pilot-conclusion-wi-state-laboratory-of-hygiene/	Mar-19	14-Jul-17	31-Jan-19
US	New York	1,702,482	55	12	55	67	122	141,874	30,954	25,410	18%	82%	New York State Newborn Screening Program	29-Jun-21	1-Oct-14	27-May-22
US	Missouri	652,374		13	49	62	62	50,183	13,314	10,522	21%	79%	Missouri Department of Health, Jefferson City, MO	17-Aug-22	Jan-13	Dec-21
US	Georgia (P)	59,332	1	1	2	3	4	59,332	29,666	19,777	33%	67%	Int J Neonatal Screen. 2020 March; 6(1). doi:10.3390/ijns6010002	1-Mar-20		
US	Ohio	797,109	6	9	31	40	46	88,568	25,713	19,928	23%	78%	Ohio Newborn Screening Program	29-Jun-22	1-Jul-16	25-Jun-22
Asia	Taiwan (SS)	1,371,857	0	20	47	72	72	62,446	26,573	19,054	28%	65%	NTUH Newborn Screening Program (unpublished), doi.org/10.1016/j.cca.2014.01.030, doi:10.1016/j.ymgme.2012.04.013, doi: 10.3390/ijns6020030	Various	Various	Various
Eu	Austria	34,736		0	4	4	4		8,684	8,684	0%	100%	DOI:10.1016/S0140-6736(11)61266-X	30-Nov-11	Jan-10	Jul-10
Eu	Hungary	40,024	3	7	2	9	12	5,718	20,012	4,447	78%	22%	DOI 10.1007/8904_2012_130	21-Mar-12		
Asia	Japan	296,759		1	7	8	8	296,759	42,394	37,095	13%	88%	https://doi.org/10.1186/s13023-021-02146-z	18-Dec-21	Apr-13	Oct-20
Eu	NE Italy (P)	44,411		0	2	2	2		22,206	22,206	0%	100%	https://doi.org/10.1007/s10545-017-0098-3	15-Nov-17	Sep-15	Jan-17
LA	Mexico	20,018				1	1			20,018			http://dx.doi.org/10.1016/j.ymgme.2017.03.001	9-Mar-17	Jul-12	Apr-16
LA	Brazil	17,716		0	3	3	3		5,905	5,905	0%	100%	Unpublished data from Brazil (Dr. Roberto GIugliani and Dr. Francyne Kubaski)	Mar-21	Jan-19	Feb-21
US	Minnesota	284,372		2	17	19	19	142,186	16,728	14,967	11%	89%	Minnesota Department of Health, St. Paul, MN	13-Jun-22	1-Aug-17	31-Dec-21
US	Kentucky	245,637		0	13	13	13		18,895	18,895	0%	100%	Kentucky Newborn Screening Program	23-Dec-20	Feb-16	Jul-20
US	Michigan	465,703		4	27	31	31	116,426	17,248	15,023	13%	87%	Michigan Newborn Screening Program, 2017-2021	17-Jun-22	Aug-17	Dec-21
US	Tennessee	425,700		5	31	36	36	85,140	13,732	11,825	14%	86%	Tennessee Department of Health, Newborn Screening Program	8-Jul-22	1-Jul-17	31-May-22
US	Pennsylvania	833,413	40	4	55	59	99	208,353	15,153	14,126	7%	93%	Pennsylvania Department of Health, Harrisburg, PA	10-Jun-22	1-Feb-16	30-Apr-22
US	Delaware	27,051		0	1	1	1		27,051	27,051	0%	100%	Delaware Division of Public Health Newborn Screening Program	29-Jun-22	1-Jan-20	15-Jun-22
US	Florida	410,681	7	2	17	19	26	205,341	24,158	21,615	11%	89%	Florida Department of Health, Newborn Screening Program, Public Records Request	29-Jun-22	3-Feb-20	31-Dec-21
US	Oregon	134,432		1	13	14	14	134,432	10,341	9,602	7%	93%	Oregon Newborn Screening Program	8-Jul-22	1-Oct-18	31-Dec-21
US	Maryland	126,762		0	5	5	5		25,352	25,352	0%	100%	Maryland Department of Health, Division of Newborn Screening	18-Mar-21	17-Jun-19	15-Mar-21
US	Virginia	350,000		0	20	20	20		17,500	17,500	0%	100%	Virginia Department of Health, Newborn Screening Program	29-Jun-22	Jan-19	Jun-22
US	Mississippi	143,219		0	8	8	8		17,902	17,902	0%	100%	MS Newborn Screen 3.0 Access Database	7-Apr-21	Jul-16	Dec-20
US	Nebraska	92,747		0	6	6	6		15,458	15,458	0%	100%	Nebraska Newborn Screening Program	29-Jun-22	1-Jul-18	31-Mar-22
US	Indiana	151,682		2	9	11	11	75,841	16,854	13,789	18%	82%	Indiana Department of Health, Newborn Screening Reports	9-Jun-22	1-Jul-20	31-May-22
US	New Jersey	283,493		0	5	5	5		56,699	56,699	0%	100%	New Jersey Department of Health	12-Jul-22	22-Jul-19	12-Jul-22
US	Washington (SS)	279,544		1	9	10	10	279,544	31,060	27,954	10%	90%	Washington State Department of Health, NBS program and pilot study data (doi:10.1016/j.jpeds.2013.01.031 & doi:10.1016/j.ymgme.2016.05.015.)	Various	Various	Various

**Table:**

Region	State/Country	*N*	Unknown	IOPD	LOPD	Total Pompe	Total Incl. unknown	IOPD Prevalence_birth_ (1:X)	LOPD Prevalence_birth_ (1:X)	Total PD Prevalence_birth_ (1:X)	% IOPD	% LOPD	Comment
US	US	9,794,141	117	63	459	522	639	155,463	21,338	18,763	12%	88%	
Asia	Asia	1,668,616	0	21	54	80	80	73,604	28,624	20,858	28%	72%	Excludes Taiwan subset 1 from IOPD, LOPD prevalence and % - data not discretized
Eu	Eu	119,171	3	7	8	15	18	17,024	14,896	7,945	47%	53%	
LA	LA	37,734	0	0	3	4	4		5,905	9,434	0%	100%	Excludes Mexico from IOPD, LOPD prevalence and % - data not discretized
**All**	**Global**	**11,619,662**	**120**	**91**	**524**	**621**	**741**	**126,118**	**21,902**	**18,711**	**15%**	**85%**	**Excludes Mexico, Taiwan subset 1 from IOPD, LOPD prevalence and % - data not discretized**

## Appendix C

Note, the scale for comparison proportion (x axis) is 20× “zoomed out” for the earlier analysis (Martiniuk, Ausems) vs. the additional resolution possible for the more recent ones (Park, NBS).

A visualization of the uncertainty range results in table 2:

## Appendix E

Updated binomial analysis of 12,060,529 newborns screened for PD with a birth prevalence of 1:18,698.
